# Visualizing the Nanoscopic Field Distribution of Whispering-Gallery Modes in a Dielectric Sphere by Cathodoluminescence

**DOI:** 10.1021/acsphotonics.3c00041

**Published:** 2023-03-15

**Authors:** Izzah Machfuudzoh, Tatsuki Hinamoto, F. Javier García de Abajo, Hiroshi Sugimoto, Minoru Fujii, Takumi Sannomiya

**Affiliations:** † Department of Materials Science and Engineering, School of Materials and Chemical Technology, Tokyo Institute of Technology, 4259 Nagatsuta, Midori-ku, Yokohama 226-8503 Japan; ‡ Department of Electrical and Electronic Engineering, Graduate School of Engineering, Kobe University, Kobe 657-8501, Japan; § ICFO-Institut de Ciencies Fotoniques, The Barcelona Institute of Science and Technology, 08860 Castelldefels (Barcelona), Spain; ∥ ICREA-Institució Catalana de Recerca i Estudis Avancats, Passeig Lluís Companys 23, 08010 Barcelona, Spain

**Keywords:** whispering-gallery mode, Si nanoparticle, Mie mode, cathodoluminescence, scanning transmission electron microscopy

## Abstract

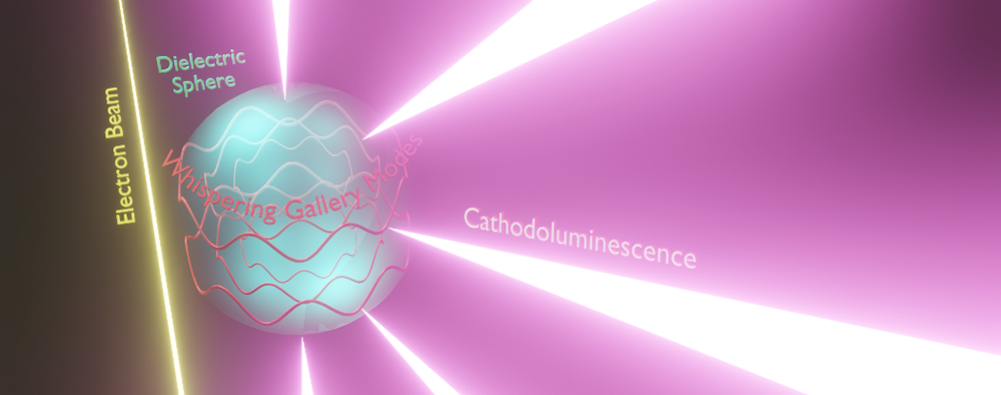

A spherical dielectric particle can sustain the so-called whispering-gallery modes (WGMs), which can be regarded as circulating electromagnetic waves, resulting in the spatial confinement of light inside the particle. Despite the wide adoption of optical WGMs as a major light confinement mechanism in salient practical applications, direct imaging of the mode fields is still lacking and only partially addressed by simple photography and simulation work. The present study comprehensively covers this research gap by demonstrating the nanoscale optical-field visualization of self-interference of light extracted from excited modes through experimentally obtained photon maps that directly portray the field distributions of the excited eigenmodes. To selectively choose the specific modes at a given light emission detection angle and resonance wavelength, we use cathodoluminescence-based scanning transmission electron microscopy supplemented with angle-, polarization-, and wavelength-resolved capabilities. Equipped with semi-analytical simulation tools, the internal field distributions of the whispering-gallery modes reveal that radiation emitted by a spherical resonator at a given resonance frequency is composed of the interference between multiple modes, with one or more of them being comparatively dominant, leading to a resulting distribution featuring complex patterns that explicitly depend on the detection angle and polarization. Direct visualization of the internal fields inside resonators enables a comprehensive understanding of WGMs that can shed light on the design of nanophotonic applications.

## Introduction

Wave fields confined within a round resonator or cavity can generate propagation modes known as whispering-gallery modes (WGMs) around the structure. The term WGMs can be traced back to the 19th century and was first proposed by Lord Rayleigh to describe a peculiar phenomenon associated with circulating acoustic waves observed in the interior of the dome of St. Paul’s Cathedral: one could hear a whisper made at the far opposite end of the dome due to how the sound waves ‘creep’ around the gallery wall.^1^ The same mechanism responsible for these whispering-gallery waves is also observed in electromagnetic waves, whereby guided light waves circulate inside and around a rounded cavity, inside of which they are confined by reflections from the concave surface of the optical resonator, and return to the original position in-phase, provided the traveled path length is equal to an integer multiple of the wavelength. Subsequently, standing waves are formed once the resonance condition is met through self-constructive interference.

The principle of WGMs holds a paramount position in practical applications and has attracted considerable interest in a number of different fields such as optical sensing,^2,3^ optoelectronics,^4,5^ optical communications,^6,7^ spectroscopy,^8,9^ and solar energy harvesting.^10,11^ For example, in highly demanded applications like sensors, the resonance frequency and linewidth of WGMs have been utilized to accurately monitor the changes occurring in a host medium, attributed to the small mode volume and strong light–matter interaction of the WGMs that enable an ultrasensitive optical detection.^12,13^ Moreover, high quality-factor (*Q*-factor) WGM resonators have also been employed in modern telecommunications that are in demand of narrow bandwidths and a high spectral density of modes that can carry and simultaneously send a number of independent signal channels, creating high-capacity communication systems.^14,15^ In other words, WGMs have been extensively studied in various contexts for their appealing characteristics of featuring high *Q*-factor, minimal mode volume, and the ability to strongly enhance light–matter interactions. However, the visualization of the fields associated with WGMs is still limited to simulations of the circulating waves, despite the fact that such field distributions are essential to investigate, customize, and apply WGMs. In this context, there are just indirect observations, such as the redistribution of the field in optical sensing by a silica microtoroid resonator when perturbed by an external molecule,^3^ or the radiation patterns of an antenna along the rim of a segmental dielectric disk resonator.^16^ Moreover, field imaging is currently limited to only externally taken photographs of the associated waves, as demonstrated by microscope images of stopped light that display the interference patterns of the field along the surface of a fused silica microsphere.^17^ It is hence worth noting that, despite the fact that the distribution of whispering-gallery fields has been used to analyze the underlying mechanisms in designing efficient WGM-based applications, an experimental acquisition of the optical fields of WGMs that directly visualize the distribution of the internal field inside a resonator has not yet been realized.

To access the WGM field, which resides mostly inside the structure or material, a probe that can reach inside, yet with nanoscopic spatial resolution, is necessary.^18^ Electron beam-based methods, such as electron energy-loss spectroscopy (EELS) and cathodoluminescence (CL), are the most relevant techniques that can effectively excite optical modes inside the samples by means of high-energy electrons, and in fact, they have been extensively utilized to study optical WGMs.^19,20^ However, in this regard, EELS is not capable of selecting the excited degenerate modes, in contrast to CL, which can be used to visualize complex mode interferences as well as to resolve even several interfering degenerate modes with the aid of light polarization filters and angle-resolved capabilities.^21,22^ Consequently, these abilities make the CL technique suitable to perform optical field visualization of mode interferences, with the measured CL signal being proportional to the radiative component of the electromagnetic local density of states (EMLDOS) along the electron propagation direction.^23^

In this work, we report on experimentally measured and selectively visualized internal field distributions of whispering-gallery modes within dielectric Si spherical resonators, excited upon electron-beam irradiation, using a measurement system capable of performing CL-based scanning transmission electron microscopy (STEM-CL). Silicon spheres were chosen because this material has a high refractive index relative to its density, and thus, the internal field is accessible in depth by accelerated electrons penetrating inside the particles, while higher-order WGMs can be addressed as well. The CL signals acquired from a dielectric sphere can be assigned to the eigenmodes of an open cavity hosting leaky whispering-gallery optical waves, which typically have lower *Q*-factors than non-radiative modes but still sustain WGM.^24^ We first demonstrate field mapping of several Si spheres with different diameters, and then perform a detailed investigation of the resolved modes of one selected sphere and the respective dominant modes extracted by resolving degeneracies using angle-, polarization-, and wavelength-resolved CL-based methods. The observed modes as well as the corresponding simulated modes can be indexed according to either WGM or Mie (multipole) terminology, indicating that both interpretations are valid. Our results on the imaging of the mode fields open the way to experimental realizations needed in optimizing WGM-based applications.

## Methods

### 4D STEM-CL Measurement System

**Figure 1:**
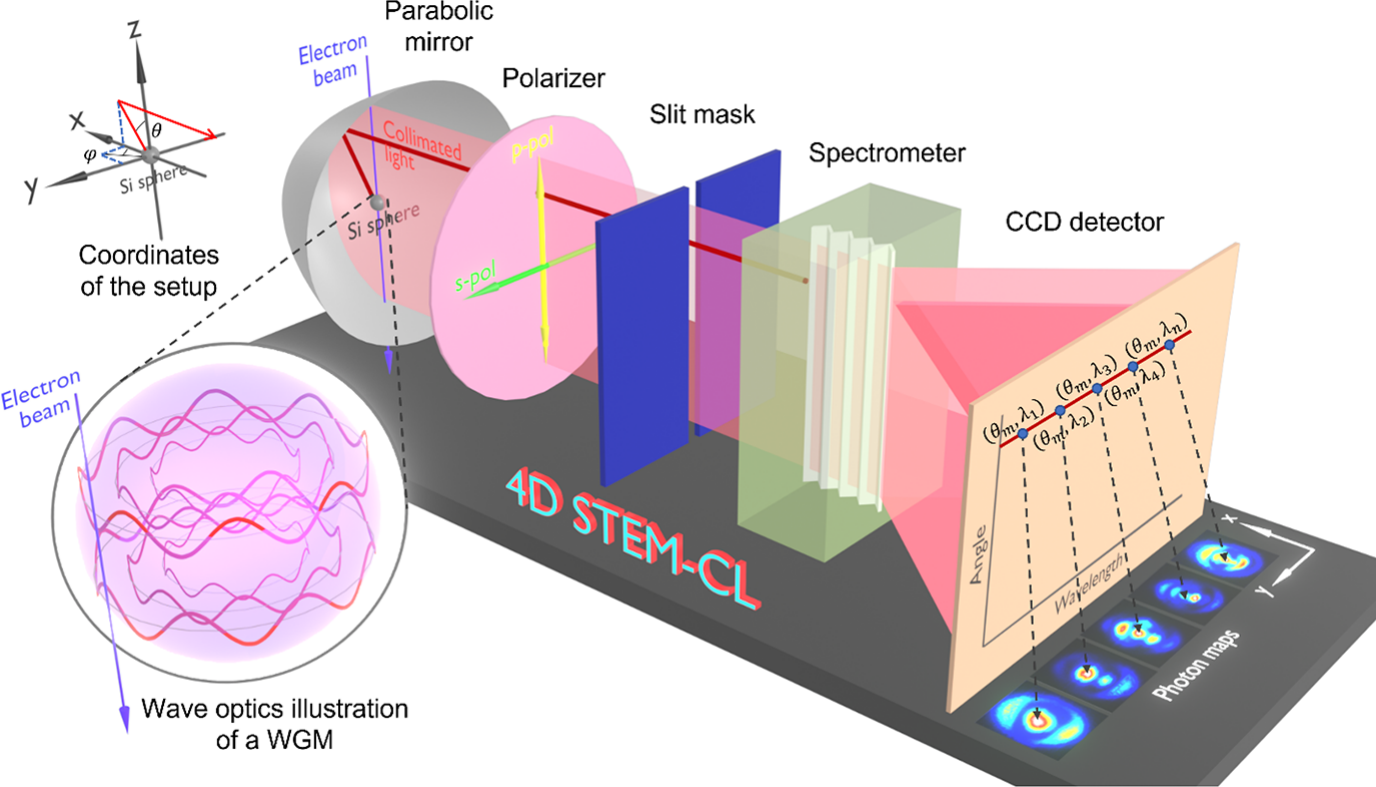
Schematic illustration of our 4D STEM-CL measurement system, with the coordinate frame referred to the experimental setup. The system allows for the acquisition of 2D photon maps at different detection angles and wavelengths, namely, (θ_*m*_, λ_*n*_), by capturing one shot to visualize the excited whispering-gallery modes inside a Si sphere upon electron-beam irradiation.

A modified instrument (JEM-2100F, JEOL) with a Cs-corrector is used as a STEM-CL measurement system. The electron beam is set with an acceleration voltage of 80 keV. An electron probe current of 1 nA and an illumination half-angle of 20 mrad are set to realize a probe size of the order of 1 nm.^21^ The CL signal that is emitted by the sample upon beam irradiation is effectively collimated from both the upper and lower sides of the sample by a parabolic mirror (Figure 1). The collimated light is then brought outside of the vacuum of the STEM instrument and made to pass through a polarizer before entering a slit mask that separates p- or s-polarization components in the emitted radiation with a fixed azimuthal angle at φ = 0^°^ while simultaneously collecting complete polar angular information in the θ angular direction. Through this setup, our STEM-CL system thus allows for the selection of the interfering degenerate modes by resolving their indices and signs.^25,26^ The resulting planar beam then hits a grating at the spectrometer before finally reaching a charge-coupled device (CCD) detector that collects the two-dimensional (2D) angle-wavelength information carried by the CL light by preserving the resolved polar angular distribution θ along its vertical axis and the dispersed wavelength λ along its horizontal axis.^27^ Details on the interpretation of data of the angle- and wavelength-dispersion patterns (the so-called angle-resolved spectrum (ARS)) can be found elsewhere.^27^ Note that we set the spectral resolution around 4 nm in order to reduce the size of the obtained 4D data set, which however is still sufficient to investigate the WGMs of the chosen particles in this study. Combined with raster scanning of the electron beam on the sample in *xy*-space, an additional two dimensions of information are available during imaging of the collected CL signal, as shown in Figure 1. The spatially resolved emission is here denoted photon map^21^ and can be extracted from the acquired 4D data set for a certain fixed detection angle θ and wavelength λ. Each image consists of 50 × 50 pixels, each with a dwell time of 1 s.

### Analytical Multipole Decomposition (AMD) Simulation

The cathodoluminescence signal emitted from a Si sphere is simulated by using an analytical multipole decomposition (AMD) method, based on an analytical multipole expression of the electromagnetic field excited by a fast electron.^27,28^ Simulations of the CL spectra as well as the CL maps are performed by setting the electron energy to 80 kV, which corresponds to the actual acceleration voltage of the electron beam used in our CL measurements. Integrated spectra are simulated by considering unpolarized CL emission over the entire range of angular directions (0^°^ ≤ θ ≤ 180^°^ and 0^°^ ≤ φ ≤ 360^°^) and over the entire particle. A sufficiently high number of multipoles are included in the simulation, limited by the maximum angular momentum number , to guarantee convergence with respect to this parameter. We also calculate angle-resolved spectra and maps by taking into account the polarization of the CL emission while fixing the polar angle θ and the azimuthal angle φ = 0^°^. Similarly, mode-resolved spectra and maps with only the selected angular mode  and the respective azimuthal numbers *m* within the range delimited by |*m*| ≤  are calculated to evaluate the contribution of each mode.

### Fabrication of Si Spheres

Silicon particles with various sizes are fabricated by thermal disproportionation of SiO into Si and SiO_2_ at a temperature higher than the melting point of the bulk Si crystal. First, SiO lumps (Wako, 99.9%) are crushed to a powder before annealing at 1550^°^C for 120 min in a N_2_ gas atmosphere.^29^ In softened SiO_2_ matrices, the annealing process produces spherical droplets from the molten Si particles that eventually grow bigger by absorbing other smaller ones. This process is followed by preserving the spherical shape of the droplets through a cooling process. After SiO_2_ is etched out in hydrofluoric acid solution (46 wt%) for 1 h, the extracted Si particles are transferred to methanol and finally subjected to ultrasonication (Violamo SONICSTAR 85) for 1 min. The oxide layer formed on the surface of the particles is of the order of 1 nm, which is too thin to produce any significant effect on the optical properties of the WGMs.^29^ Solutions of Si nanoparticles are then dropped onto elastic carbon grids with a thickness of around 20 nm, which have a minimal influence on the mode energies and fields for spherical particles.^30^

## Results and Discussion

### WGMs for Different Si Sphere Sizes

**Figure 2:**
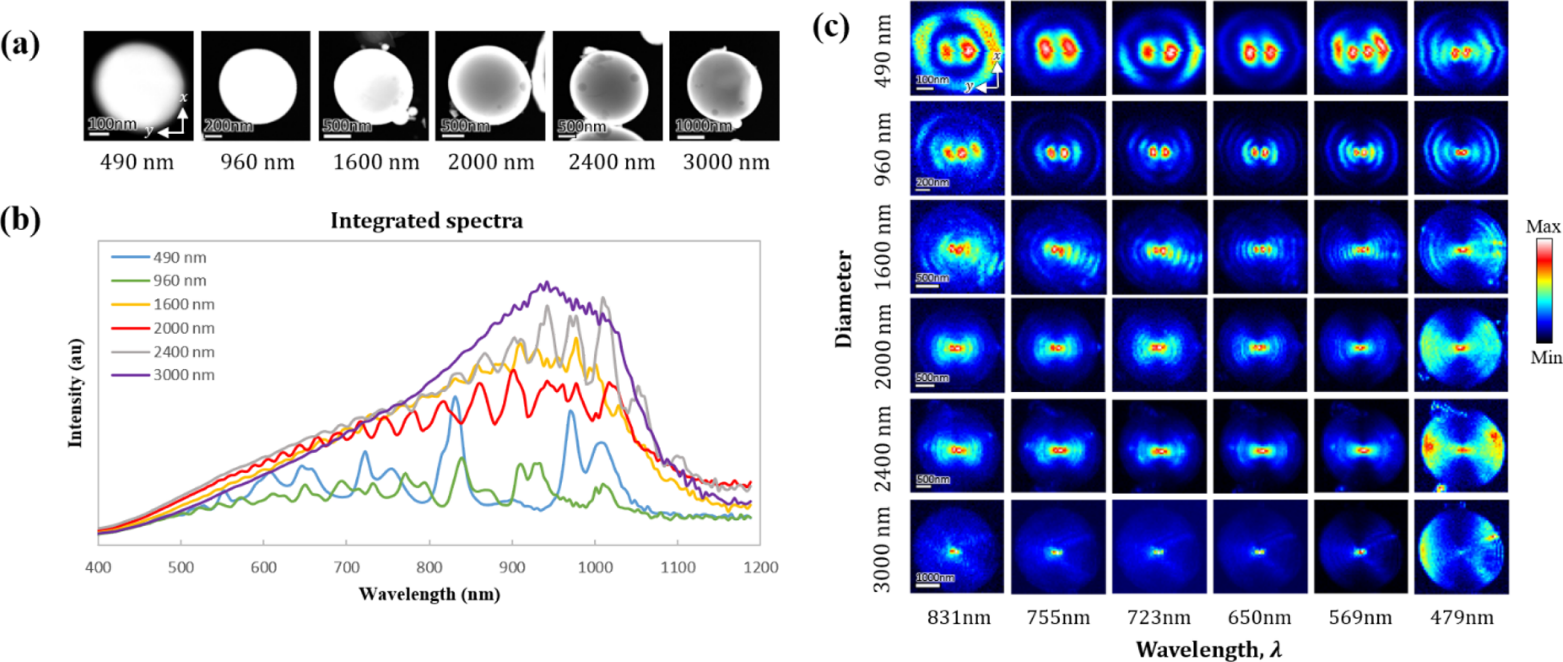
WGMs in Si spheres of different diameters. (a) Dark-field STEM images with their diameters indicated at the bottom, together with the scale bar. The spatial *xy* coordinate frame shared by all spheres is shown in the first image. (b) Measured unpolarized CL spectra of the observed Si spheres with the CL signal integrated over an electron beam scan of the entire particle. (c) Photon maps of the Si spheres shown in panel (a) at some representative wavelengths, taken at a detection angle θ = 180^°^ for s polarization.

Silicon particles with various diameters are measured to study the relation of the sphere sizes with the existing WGMs. Figure 2a shows the dark-field STEM images of Si spheres with diameters ranging from 490 to 3000 nm. The measured unpolarized CL spectra that integrate the signal by averaging the beam position over the entire particle are shown in Figure 2b. Each spectrum contains a series of discrete resonances that can be understood to arise due to light guiding within the sphere, circulating a number of revolutions and returning in phase with constructive interference, thus resulting in the formation of resonant standing waves.^31^ Such an optical mechanism gives rise to the presence of WGMs in the Si spheres. We note that the observable WGMs are leaky modes since far-field signals are detected in the CL system,^32^ and in addition, the presence of a surface layer of inclusions on the Si spheres could perturb the optical properties of the WGMs, possibly affecting their *Q*-factors.^33^ Moreover, there is a drop in spectral intensity around a wavelength of 1000 nm due to the sensitivity of the spectrometer camera. The dependence of the CL spectra on the sphere sizes (see Figure 2b) indicates that, as the diameter of the particle increases, the total number of peaks present in the spectrum increases too, meaning that larger particles can accommodate more resonance modes inside them.^34^ In essence, for a fixed value of the permittivity, the mode structure is a function of the light wavelength normalized to the particle radius, and therefore, considering the spectral region determined by a given lower value of the wavelength, when increasing the radius, one is essentially moving an increasing number of modes toward that region. Figure 2c shows maps for the internal field distribution of the WGMs produced by emitted CL light that is collected at a detection angle θ = 180^°^ with s polarization. Under this configuration, a symmetric pattern along the *x* axis is observed with strong hotspots at the center surrounded by layers of ring-shaped hotspots extending toward the edge of the particle in the radial direction. It is observed that for a given Si sphere with some fixed diameter, the number of ring-shaped hotspots increases as the wavelength decreases. This shows that the particle can indeed accommodate higher-order modes at shorter wavelengths. Similarly, for a given wavelength λ, the number of layered ring-shaped hotspots is also observed to increase sharply as the particle size increases, indicating that larger particles can indeed sustain higher-order modes than the smaller ones. As a result, a vast number of ring-shaped hotspots in the larger particles are consequently formed and closely packed fringe patterns appear, for example, in the particle with a diameter of 3000 nm at a wavelength of 479 nm in Figure 2c. However, due to the sustained modes that are spectrally and spatially overlapped with each other, the fringe patterns in larger particles are difficult to be resolved or analyzed. Therefore, we choose a smaller particle with a diameter of 490 nm for a detailed discussion in the following sections (i.e., a particle size for which the noted ratio of wavelength to radius lies within an interesting range of the experimentally accessible spectral region), for which the particle still accommodates WGM resonances with periodic peaks in the spectrum and a distinctively observable distribution of hotspots in the photon maps.

### Assignment of WGM Index

**Figure 3:**
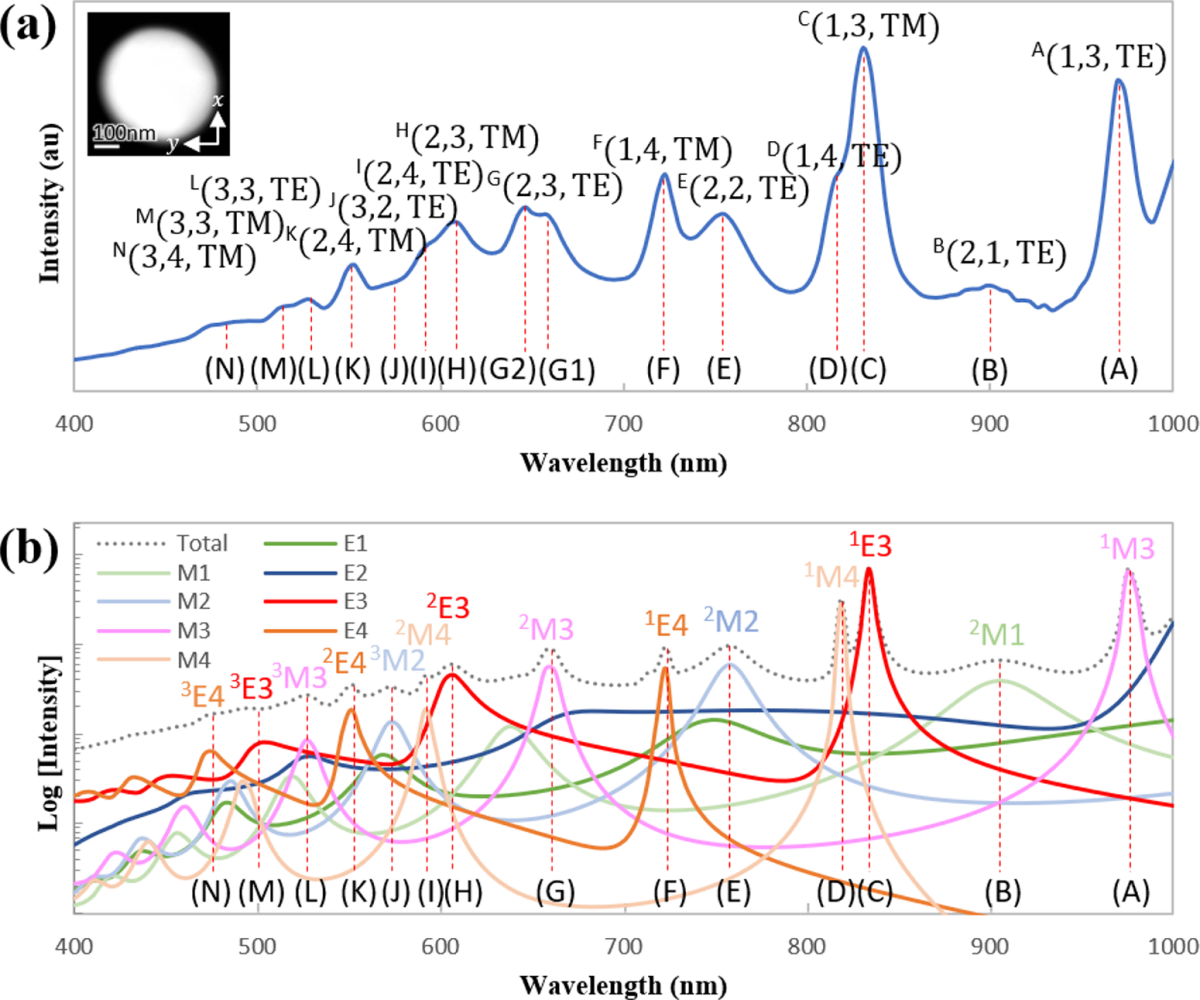
Detailed spectral mode analysis of a representative particle. (a) Experimentally measured unpolarized CL spectrum for a Si sphere of 490 nm in diameter, with the inset showing the STEM dark-field image of the corresponding particle. The obtained CL signal is integrated over the entire particle and all emission angles 0^°^ ≤ θ ≤ 180^°^. Labels on the resonance peaks refer to the WGM index with parameters (*n*, , *P*). The superscripts on the left side correspond to the peak labels. (b) Simulated integrated CL spectrum of a Si sphere that includes all possible excited modes (dotted line) and also only one extracted mode (colored curves). Labels on the resonance peaks refer to the modes with the notation ^n^. Parameters used in both WGM and multipole expansions preserve the same descriptions of radial order *n*, angular momentum number , and polarization state *P* as the field oscillation.

The experimentally obtained unpolarized spectrum for the Si sphere with a diameter of 490 nm is shown in Figure 3a with the resonance peaks labeled alphabetically from #A to #N to facilitate the discussion. The collected CL signal is spatially integrated over the whole particle area with a detection angle θ that covers the entire polar direction from 0^°^ to 180^°^ to include all the possible modes that are excited upon beam irradiation on the sphere. The azimuthal angle is fixed at φ = 0^°^. The observed resonances are compared to the spectrum by AMD theory, as shown in Figure 3b, in which the dotted line refers to the unpolarized integrated spectrum that includes all the excited modes, while the colored solid lines refer to the mode-selected spectra that include only the extracted mode. Mode labeling in the multipole expansion of the AMD simulation is indicated by a three-term label as ^n^, in which the uppercase *P* corresponds to either a magnetic mode *M* or an electric mode *E*, and the parameter  denotes the multipole order (i.e., the total angular momentum number) with  = 1 for dipoles,  = 2 for quadrupoles, and so on. Owing to the properties of the dielectric particles that can accommodate electric fields inside the particle,^26^ the presence of multiple field antinodes in the radial direction allows us to define a radial order *n* that is indicated as a superscript in the mode label. Each resonance peak is contributed by multiple modes. However, it is generally possible to extract one mode with the highest relative intensity (i.e., a dominant mode that contributes the most signal to the observed resonance). Such a dominant mode in the multipole expansion is shown for every peak in Figure 3b through the ^n^ label, together with the #A–N tags corresponding to the experimental results. While the measured spectrum shows good agreement with the calculated one, the experimental peak #G is observed to be split into two peaks (#G1 and #G2, respectively). This could be due to a geometrical deviation of the particle shape from a perfect sphere, which could remove mode degeneracy, as will be discussed later together with the mapping of field profiles.

Through the relation of the electromagnetic fields of a dielectric sphere to the WGM conditions expressed in terms of Bessel functions and Legendre polynomials that respectively determine the radial and angular orders of the wave,^35^ the aforementioned three indices *n*, , and *P* in the multipole expansion that describe the electromagnetic eigenmodes of a dielectric sphere can be proportionally translated to the parameters that explicitly characterize the whispering-gallery modes in the WGM expansion. For the mode index *P*, the electric mode *E* in the multipole expansion refers to the transverse magnetic (TM) mode in the WGM expansion for the radially polarized electric field, and vice versa, the magnetic mode *M* for an azimuthally polarized electric field of the transverse electric (TE) mode. Resonance peaks in the WGM expansion are therefore identified as (*n*, , *P*), as labeled in Figure 3a. The superscripted character on the left side of the WGM index refers to the labels of each peak. Moreover, an identification of the WGM index directly from the WGM expansion is also performed through the theoretical calculation of the approximate positions of the resonances by considering the argument of the Bessel function with the fields near the resonator surface,^36^ and the results excellently match with the experimental ones (see Table S1 in the Supporting Information (SI) for more details). We observe that, for a given field polarization *P*, the radial order *n* increases as the wavelength decreases, and so does the angular momentum order  for a given set of parameters *P* and *n*. In physical terms, this means that, at shorter wavelengths, resonances with higher radial order *n* accommodate more antinodes along the radial direction, and so do the higher angular modes  along the polar direction by also depending on the azimuthal numbers *m* that determine the distribution of the resonant mode field in the azimuthal direction.^37^ The former interpretation can be observed spatially as photon maps shown in Figure 2c, in which at shorter wavelengths the field intensity is more concentrated around the center of the sphere for a given particle size through the presence of many layers in the ring-shaped hotspots. Again, this supports the idea of how a dielectric particle could accommodate higher-order modes at shorter wavelengths, as discussed above. Likewise, lower-order modes with small *n* and high  are not dominantly observed because they become less radiative and, therefore, hidden from CL detection. Field visualization projected as photon maps at resonance peaks with wavelength-dispersed and angle-resolved elements for a given polarized light component are discussed in more detail in the next section.

### Field Distribution with a Single Dominant Mode

The distribution of the electric field in a dielectric sphere can be visualized by raster scanning the electron beam over the particle. Angle-resolved mapping, in which the emitted light is collected at a certain detection angle, allows for the observation of the resolved modes as well as the corresponding dominant mode that is selected out of their degeneracy. By combining the polarization- and wavelength-resolved capabilities, the distribution of the internal nanoscopic hotspots for the excited WGMs can be selectively extracted. For the simulated CL maps, we shall use the term ‘all-mode mapping’ for field visualization including all the possible existing modes, and the term ‘dominant-mode mapping’ for imaging of only one dominant mode.

**Figure 4:**
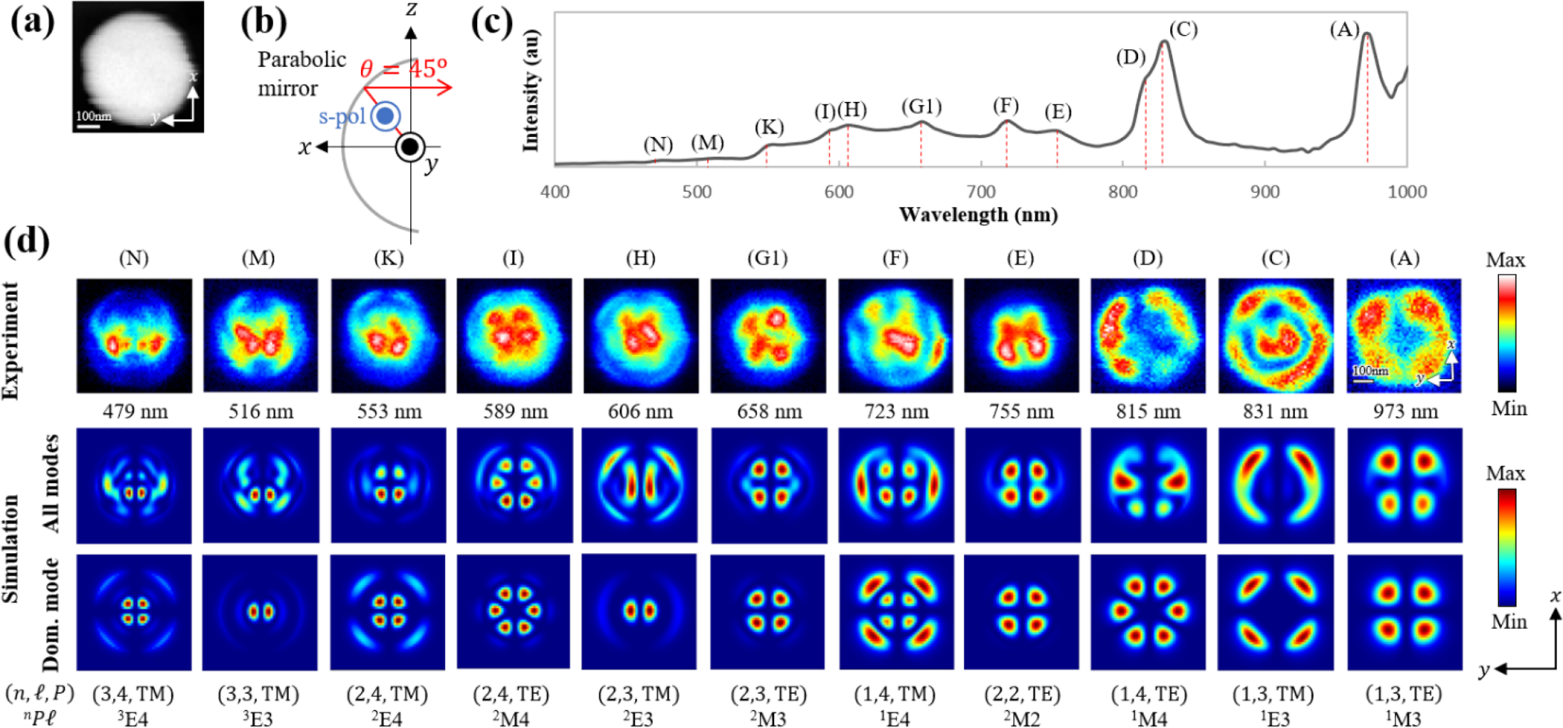
Field distributions under conditions such that a single mode dominates the signal. (a) STEM image of a Si sphere with a diameter of 490 nm, obtained simultaneously with photon mapping. (b) Detection configuration of the measurement system for s-polarized light at an emission angle (θ, φ) = (45^°^,0^°^). (c) Measured CL spectrum integrated over the entire particle, detected at an angle θ = 45^°^. (d) Photon maps of the corresponding Si sphere. The top, middle, and bottom rows show the obtained experimental maps, the simulated all-mode maps, and the dominant-mode maps, respectively. Resonance wavelengths for each peak are indicated below the experimental maps, with the WGM index (*n*, , *P*) and the mode label ^n^ shown at the bottom.

To study the resulting field distribution, we first analyze the mapping results of s-polarized CL light emitted at the detection angle θ = 45^°^, which turns out to be largely dominated by only one single mode. Photon maps of these resonance peaks are listed in Figure 4. Peaks #A and #C at longer wavelengths of 973 and 831 nm, respectively, are shown to have four hotspots that are nicely reproduced by the simulated all-mode mappings with an asymmetric intensity distribution along the *x* direction. These hotspots are contributed by the dominant  = 3 mode with azimuthal numbers *m* = ± 2, in which the fields of the four lobes of the mode are mapped (a list of the radiation distribution pattern can be found in Figure S1 in the SI). Note how peak #A has its hotspots located more toward the interior of the sphere, while for peak #C, the hotspots are located along the particle edge instead. This is attributed to the induced electric field of the magnetic ^1^M3 mode for peak #A that is circulating inside the sphere, while the electric ^1^E3 mode for peak #C has its electric field oscillating slightly outside the particle, as observed for transverse electric and transverse magnetic modes, respectively. Regarding the two hotspots at the center of the particle for peak #C, which is also shown in the simulated all-mode mapping, it is understood to be contributed by the slightly less-dominant degenerate electric ^1^E3 mode with *m* = ± 1 (see the SI for the radiation patterns of the degenerate multipole modes). As for peak #D at a wavelength of 815 nm, the contribution from the dominant magnetic ^1^M4 mode (*m* = ± 3) produces six hotspots at an emission angle of 45^o^ that comes from the top six field lobes of the mode that are as well located at approximately the same angle position. However, due to mode interference with peak #C, these hotspots are observed to smoothly merge along the edge of the sphere as shown in both simulated and experimental all-mode mappings of resonance peak #D. Such a similar merging pattern is also observed in peak #C.

Mappings at wavelengths of 755, 723, and 658 nm, which respectively correspond to peaks #E, #F, and #G1, have their field distributions with strong four-fold symmetric hotspots inside the sphere, which are contributed by the same azimuthal *m* = ± 2 components. This can be understood from the total number of azimuthal maxima, which is 2*m*, thus resulting in four hotspots along the φ direction,^38^ as observed in the photon maps. Moreover, the contribution from the radial modes is also apparent here; for instance, *n* = 2 for peak #E with a dominant magnetic ^2^M2 mode displays two antinodes of the standing waves that are projected in the map as a field intensity with two radial-directional hotspots. In addition, for peak #F, which is dominated by the electric ^1^E4 mode, such a field distribution observed in the experimental map could be attributed to the interference with another second dominant ^2^E2 mode (see Figure S2a in the SI). Similarly, two internal weak hotspots observed in the peak #G1 mapping that are located right next to the aforementioned four strong hotspots are also due to mode interference, in which the degenerate magnetic ^2^M3 mode (*m* = ± 3) whose six hotspots are overlapped with the former four hotspots from the dominant *m* = ± 2 components gives rise to such a final pattern in the photon map.

The base pattern for the peak #H mapping at a wavelength of 606 nm is clearly dominated by the electric ^2^E3 mode (*m* = ± 1) with two hotspots located at the center and along the edge of the particle, as can be seen in the resemblance of the hotspot distribution between the all-mode and the dominant-mode mappings that are also reflected in the experimental mapping. The out-of-plane electric charges of the ^2^E3 mode with *m* = ± 1 have oscillating electric fields on the *yz*-plane, materializing the interaction between the *z*-oriented electron beam with the *z* component of the top field lobe for the two center hotspots. The beam-field interaction from the side lobes gives rise to additional two ring-like hotspots along the particle edge. Furthermore, a nearly equal contribution from the degenerate electric ^2^E3 mode (*m* = ± 2) results in the ring-like hotspots that are seen to be grouped into four parts. Similarly, overlapping with the other four internal hotspots from the same degenerate *m* = ± 2 electric mode yields the elongated two central hotspots, as can be seen in the experimental mapping. At a wavelength of 589 nm, the resonance at peak #I is dominated by the magnetic ^2^M4 mode (*m* = ± 3), which features six strong hotspots that are retained in the resulting mappings.

As the wavelength decreases, the hotspot distribution becomes more complex due to the contributions from higher-order modes. For peak #K at a wavelength of 553 nm, the electric ^2^E4 components (*m* = ± 2) is responsible for the four strong hotspots around the center of the particle. At the same time, the ring-like hotspots that are seen to be grouped into six parts are formed as a result of the six hotspots from the slightly less dominant degenerate electric ^2^E4 mode (*m* = ± 3) that overlaps with the four ring-like hotspots from the former *m* = ± 2 components. A similar grouping pattern with a four-fold feature is also observed at resonance peak #M at a wavelength of 516 nm due to the contribution from the less dominant electric ^3^E3 mode (*m* = ± 2), as indicated by the resulting mappings with four hotspots along the particle edge and also around the center of the sphere. In addition, the two strong central hotspots are attributed to the dominant degenerate electric ^3^E3 mode (*m* = ± 1); these hotspots are slightly displaced downward (along the negative *x* direction) due to the mode interference with neighboring peaks. Such a pattern of hotspot distribution is clearly shown in the experimental mapping, which agrees well with the simulated all-mode mapping. Lastly, a similar explanation of the overlap of the degenerate electric ^3^E4 mode between the dominant *m* = ± 2 and the less-dominant *m* = ± 3 components is also applicable to the peak #N mapping at a wavelength of 479 nm. Their interference leads to field features with four hotspots near the center of the particle, with the bottom two spots having stronger intensity, surrounded by six internal hotspots around it and another six ring-like hotspots along the edge of the sphere. The complementary spectra of the dominant modes at an explicit angle θ = 45^°^ with s-polarization light collection are shown in Figure S2a in the SI.

To summarize, the resulting field patterns of the resonance modes with s-polarized light at an emission angle θ = 45^°^ are observed to roughly retain the main hotspot distribution of the respective dominant modes due to their significantly strong influence.

### Field Distribution with Interfering Multiple Dominant Modes

Now, we demonstrate mappings of more complex field features stemming from the interference of multiple modes, in which the resulting field distributions are no longer dominated by just a single mode. We choose p polarization emission and an almost horizontal detection angle for the mapping, so that more modes interact with the incident electron traveling along the *z* direction; the electric field oscillation along the *z* direction tends to produce radiation parallel to the *xy* plane. Detection at the exact horizontal angle of θ = 90^°^ is practically not possible due to the shadowing effect produced by the sample support on the *xy* plane.^25^ Therefore, a slightly elevated angle θ = 80^°^ near the horizontal plane is chosen to circumvent the problem of radiation shadowing, yet with a sufficient signal from near-horizontal emission.^21,39^

**Figure 5:**
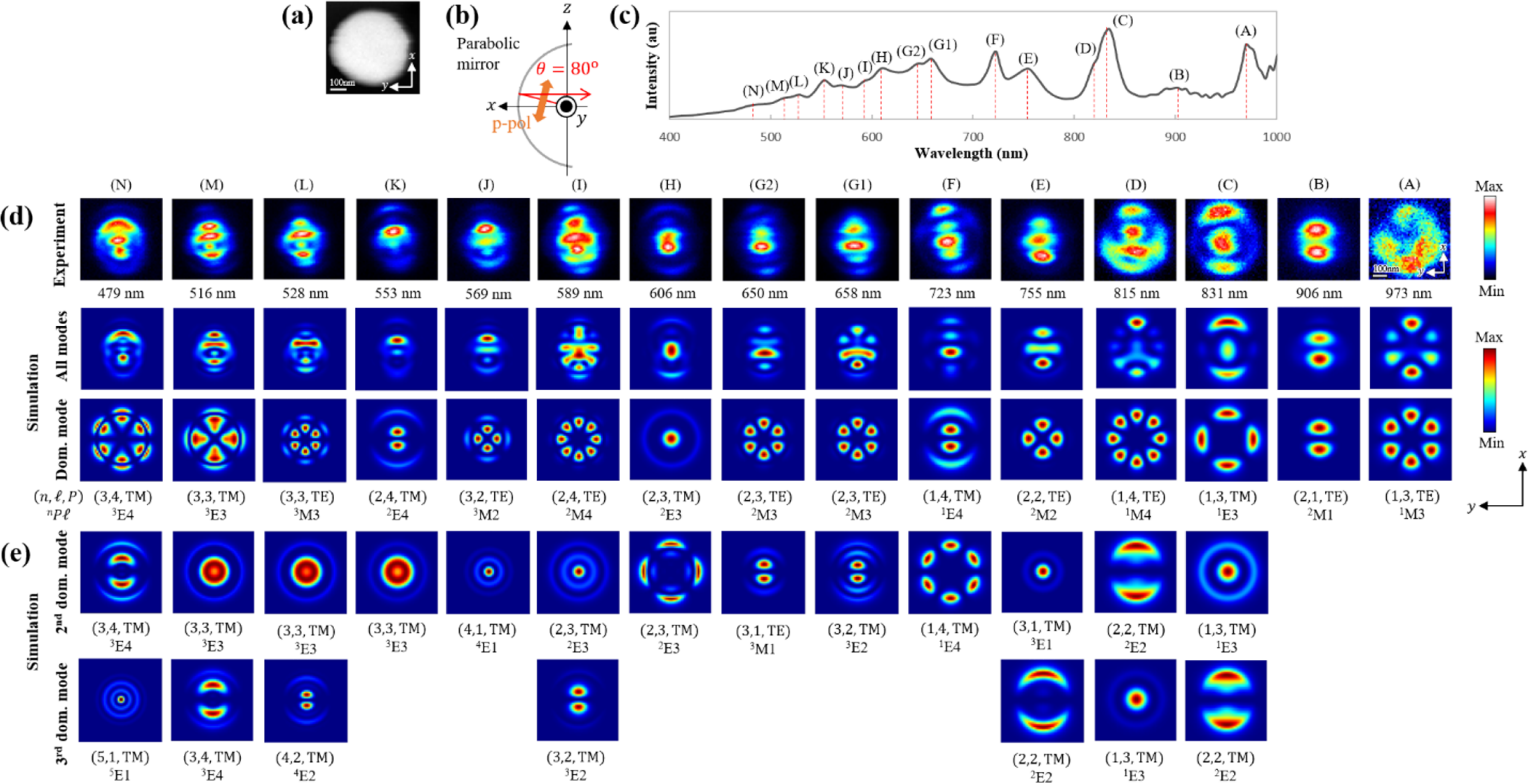
Field distributions contributed by multiple dominant modes. (a) STEM image of a 490 nm-diameter Si sphere simultaneously acquired during CL mapping. (b) Schematic illustration of the measurement condition with p polarization and a detection direction (θ, φ) = (80^°^,0^°^). (c) Experimental CL spectrum obtained by integrating over the entire particle, for emission detected at an angle θ = 80^°^. (d) p-polarized photon maps corresponding to each peak in the spectrum. The first row indicates the experimental maps with the resonance wavelengths shown below each plot, while the second and third rows display the simulated all-mode maps and the first-dominant-mode maps. The WGM index (*n*, , *P*) and mode label ^n^ are also indicated at the bottom. (e) Simulated maps corresponding to the 2nd and 3rd dominant modes.

Figure 5 shows photon maps of the same 490 nm-diameter Si sphere in this detection configuration. First, peaks #A and #B at longer wavelengths of 973 and 906 nm, respectively, are observed to nicely retain the original main hotspot distribution from their dominant mode. For instance, resonance peak #A is seen to maintain six hotspots from the dominant magnetic ^1^M3 mode (*m* = ± 3) along the particle edge, and so does peak #B by the magnetic dipole ^2^M1 mode (*m* = ± 1) with two hotspots at the center of the particle, as shown by the simulated all-mode mappings. Such distributions are also clearly reflected in the experimentally obtained photon maps.

For shorter wavelength resonances, contributions arising from the 2nd and 3rd dominant modes become significantly apparent. Their interference alters the final hotspot distributions of resonances from the main mapping pattern of the 1st dominant mode. For instance, the resonance peak #C at 831 nm has the base pattern of four hotspots along the particle edge from the 1st dominant electric ^1^E3 mode (*m* = ± 2); however, interference with the 2nd dominant degenerate *m* = 0 mode results in the appearance of a hotspot at the center of the particle, as shown by both simulated and experimental mappings. Moreover, the stronger intensities along the *x* direction are attributed to the influence of the 3rd dominant electric ^2^E2 mode (*m* = ± 1), whose hotspot distribution is aligned along the *x* axis. As a result, the final pattern of peak #C contains three hotspots aligned along the *x* axis. Similarly, the mapping pattern of peak #D at 815 nm is also formed due to the interference of the 1st dominant magnetic ^1^M4 mode (*m* = ± 4) with a nearly equal contribution from the spectrally broad peak produced by the 2nd dominant electric ^2^E2 mode (*m* = ± 1), as well as with an overlapped tail of the 3rd dominant electric ^1^E3 mode (*m* = 0) (see the mode contribution in the spectrum in Figure 3b in the main text and Figure S2b in the SI). This leads to a similar mapping to the case of the previous peak #C, but with the eight hotspots (located along the particle edge) that serve as the base pattern of peak #D mapping, although these eight hotspots are not clearly separated in the experiment, which is possibly due to the imperfection of the spherical shape as well as to the signals that are integrated over a finite angular range.

As for peak #E at 755 nm, the contribution order of the dominant modes is different for the integrated case (Figure 3b) and for the angle- and polarization-resolved one discussed here (Figure S2b in the SI). According to the latter spectra, the 3rd dominant electric ^2^E2 mode (*m* = ± 1), whose fields are circulating near the particle edge, contributes to the presence of the faintly weak hotspots at the edge of the sphere, as can be seen in both experimental and all-mode mappings. At the same time, interference between the most dominant magnetic quadrupole ^2^M2 mode (*m* = ± 2) and the 2nd dominant electric dipole ^3^E1 mode (*m* = 0) leads to the appearance of a long horizontal hotspot at the center of the particle; four hotspots from the former magnetic mode overlap with the central hotspot from the latter electric mode, as can be observed in the experimental mapping. At a wavelength of 723 nm, the resonance of peak #F that is dominated by the electric ^1^E4 mode (*m* = ± 1) and its overlap with the degenerate *m* = ± 3 components, leading to a final mapping that contains two central hotspots surrounded by six hotspots aligned at the particle edge.

Peak #G is simulated to be dominated by the magnetic ^2^M3 mode (*m* = ± 3) with the six hotspots attributed to the radiation from an in-plane magnetic pole distribution (see Figure S1). However, as a consequence of the imperfect spherical shape of the Si particle, the geometric symmetry is possibly broken and hence causes the degeneracy of this *m* = ± 3 mode to be resolved into different resonance energies. As a result, peak splitting occurs and different peak #G1 at a wavelength of 658 nm and peak #G2 at 650 nm are observed. This leads to the former peak being more influenced by the electric quadrupole ^3^E2 mode (*m* = ± 1), while the latter one interferes mostly with the magnetic dipole ^3^M1 mode (*m* = ± 1) as their 2nd dominant mode. The resulting field distribution of these mode interferences is respectively projected by the experimental mappings of peaks #G1 and #G2 shown in Figure 5. For peak #H at a wavelength of 606 nm, the strong central hotspot that is contributed by the dominant electric ^2^E3 mode (*m* = 0) interfering with the degenerate *m* = ± 2 components leads to the presence of several weak hotspots along the edge of the particle, as can be evidently spotted in the resulting mappings. As for peak #I at a wavelength of 589 nm, the 1st dominant magnetic ^2^M4 mode (*m* = ± 4) provides the base pattern with eight hotspots along the particle edge. However, their overlap with the electric ^2^E3 mode (*m* = 0) from the neighboring peak eventually generates a strong hotspot at the center of the aforementioned eight edge hotspots. Furthermore, interference with the 3rd dominant electric ^3^E2 mode (*m* = ± 1) has also altered the overall field distribution of the modes, leading to a stronger hotspot intensity observed at the bottom part of the particle. This mechanism can be attributed to the addition of the fields of equal signs resulting in more radiations being emitted at the lower side of the sphere.^40^ A similar explanation of mode interference is also applicable to peak #J at a wavelength of 569 nm, featuring an interference between the magnetic ^3^M2 mode (*m* = ± 2) and the electric ^4^E1 mode (*m* = 0), as well as to peak #K at 553 nm, which has an overlap of the electric ^2^E4 mode (*m* = ± 1) with another electric ^3^E3 mode (*m* = 0).

The shorter the wavelength, the more complicated the field pattern is. Peak #L at 528 nm is investigated to be dominated by the magnetic ^3^M3 mode (*m* = ± 3) that provides the base pattern with six hotspots. However, their interference with the 2nd dominant electric ^3^E3 mode (*m* = 0) and the 3rd dominant electric ^4^E2 mode (*m* = ± 1) has created a resulting mapping with some asymmetric intensity distributions that can be attributed to the similar reasoning to the case of peak #I, but with a flipped sign in the electric fields of the E2 mode, which then leads to a stronger intensity at the top part of the particle instead. Again, a similar understanding of the uneven distributions in the sphere can be used to explain the hotspot features of peak #M at a wavelength of 516 nm, which is dominantly composed of the contribution from electric ^3^E3 mode (*m* = ± 2), its degenerate *m* = 0 mode, and also another electric ^3^E4 mode (*m* = ± 1) that respectively serve as the 1st, 2nd, and 3rd dominant modes. Lastly, for peak #N at 479 nm, interference between the 1st dominant electric ^3^E4 mode (*m* = ± 3) and the degenerate *m* = ± 1 components results in the upper hotspots from the former mode merging along the ring-shaped direction from the latter mode, and likewise the lower hotspots. However, as a result of further interference with the 3rd dominant electric ^5^E1 mode (*m* = 0), the final hotspot distribution creates higher intensity at the top part of the particle due to, again, the constructively added fields of the equally signed field lobes. This then explains the origin of such a field feature with an unbalanced radiation distribution as observed in the experimental mapping.

In summary, hotspot distributions of the photon maps for fields that are emitted at an approximate horizontal detection angle of θ = 80^°^ with p polarization are formed as a result of interference of several modes, leading to final mapping patterns that remarkably deviate from the base pattern of their respective 1st dominant mode. The origin of such field features can be explained only by such a mode-decomposed spectral analysis instead of just by using integrated spectra or mapping.

### Resolving Degenerate WGMs at Different Detection Angles

**Figure 6:**
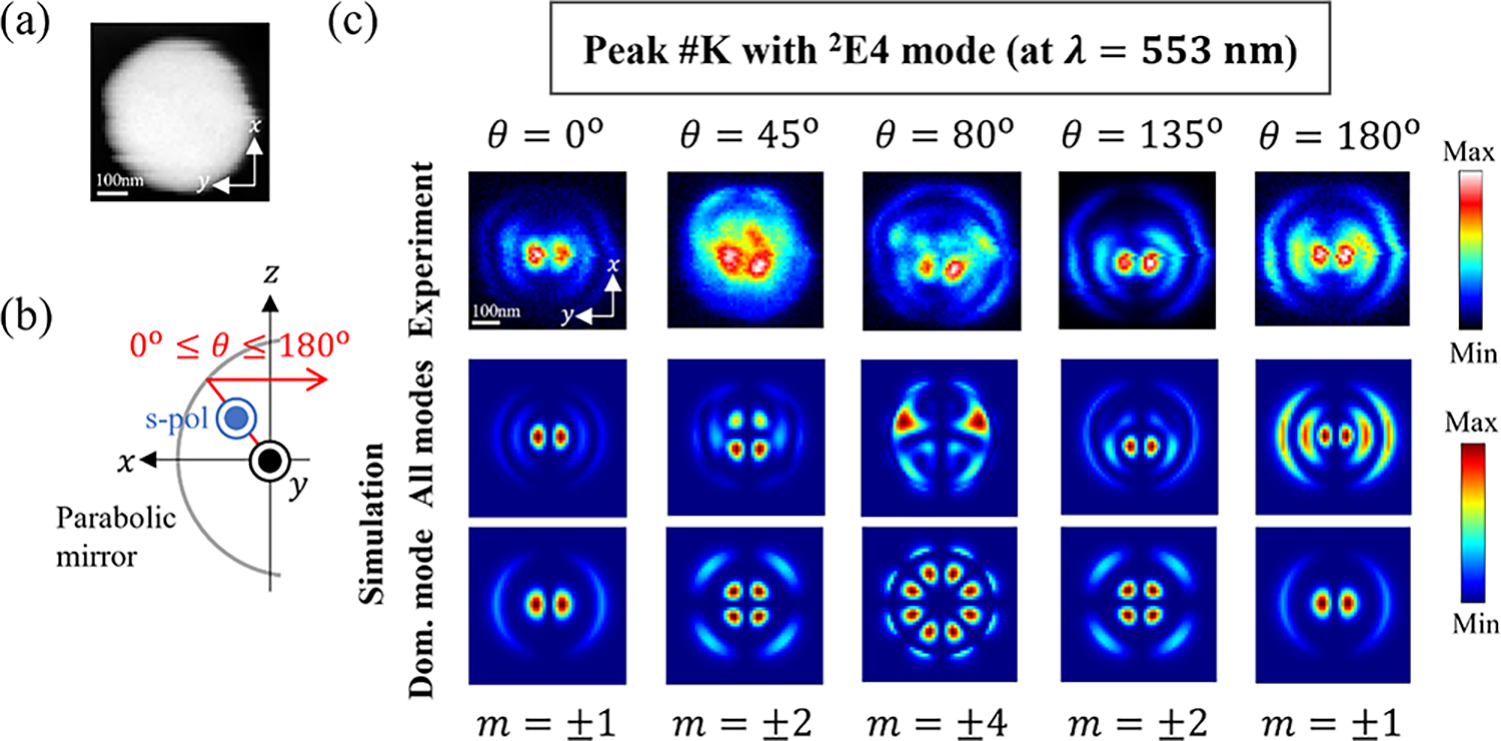
Degeneracy-resolved maps at different emission angles. (a) STEM dark-field image of a Si sphere with a diameter of 490 nm simultaneously scanned during photon mappings. (b) Setup of the system for detection of s-polarized light along a polar angle θ at a fixed azimuthal angle φ = 0^°^. (c) Experimental and simulated photon maps of peak #K with a dominant ^2^E4 mode on resonance at a wavelength of 553 nm, observed at all detection angles θ = 0^°^, 45^°^, 80^°^, 135^°^, 180^°^. The corresponding dominant azimuthal *m* components at a given angle θ are indicated at the bottom.

To further analyze mode interference in the WGM resonances, we here simultaneously compare the field mappings at different detection angles θ, as schematically shown in Figure 6b, which allows us to resolve degenerate WGMs with different *m* numbers. For a given wavelength (photon energy), photon maps are analyzed at different polar angles, namely, θ = 0^°^, 45^°^, 80^°^, 135^°^, and 180^°^, as shown in Figure 6c. We choose peak #K at a wavelength of 553 nm, which is dominated by the electric ^2^E4 mode and possesses different *m* components with distinguishable field patterns. Also, s polarization is chosen because mode interference is then weaker, as discussed above.

First, emission at an angle θ = 0^°^ is observed to have contributed the most by azimuthal numbers *m* = ± 1, as can be seen by how the resulting field distribution largely maintains the original basic pattern of the dominant *m* = ± 1 components with two central hotspots surrounded by ring-shaped hotspots. This can be deduced from the perspective of the angular radiation plots in which only *m* = ± 1 alone sustains the upward emission at θ = 0^°^ and, hence, interference with different degenerate *m* components (*m* ≠ ± 1), whether contributions from the dominant  = 4 of ^2^E4 for peak #K or any other  modes, are not expected at this angle (see Figure S1 in the SI). This analysis also applies to downward radiation at the bottom angle θ = 180^°^.

As for the inner angles at θ = 45^°^, 80^°^, 135^°^, degenerate modes with larger *m* numbers dominate the resulting field mappings. Emission at θ = 45^°^ for peak #K is calculated to be dominated mainly by *m* = ± 2 components with four hotspots at the center and edge of the particle, as shown by the dominant-mode mapping in Figure 6c. However, the contribution from other degenerate ^2^E4 modes or other off-resonance modes such as quadrupole E2 and hexapole E3 modes (see Figure S2a in the SI) produces non-negligible radiation around an angle θ = 45^°^ (see Figure S1 in the SI). Therefore, the final mappings are produced as a result of the interference of all these modes, as shown by the experimental mappings that match well with the simulated all-mode mappings in Figure 6c. Emission at an angle θ = 135^°^ can also be explained similarly, and so is the one at θ = 80^°^ with *m* = ± 4 components, which serves as a basic pattern for the in-plane radiation.

Thus, it is revealed that *m* = ± 1 can be separately observed from other degenerate modes by selecting upward and downward emission directions, while radiation collected at intermediate angles is composed of interference from several degenerate *m* components, although a dominant mode is still discernible. The 4D STEM-CL technique is thus a powerful approach to analyze and resolve such degenerate modes, since maps for all angles θ and wavelengths λ are acquired within a one-shot measurement.

### Directionality of the Radiation

**Figure 7:**
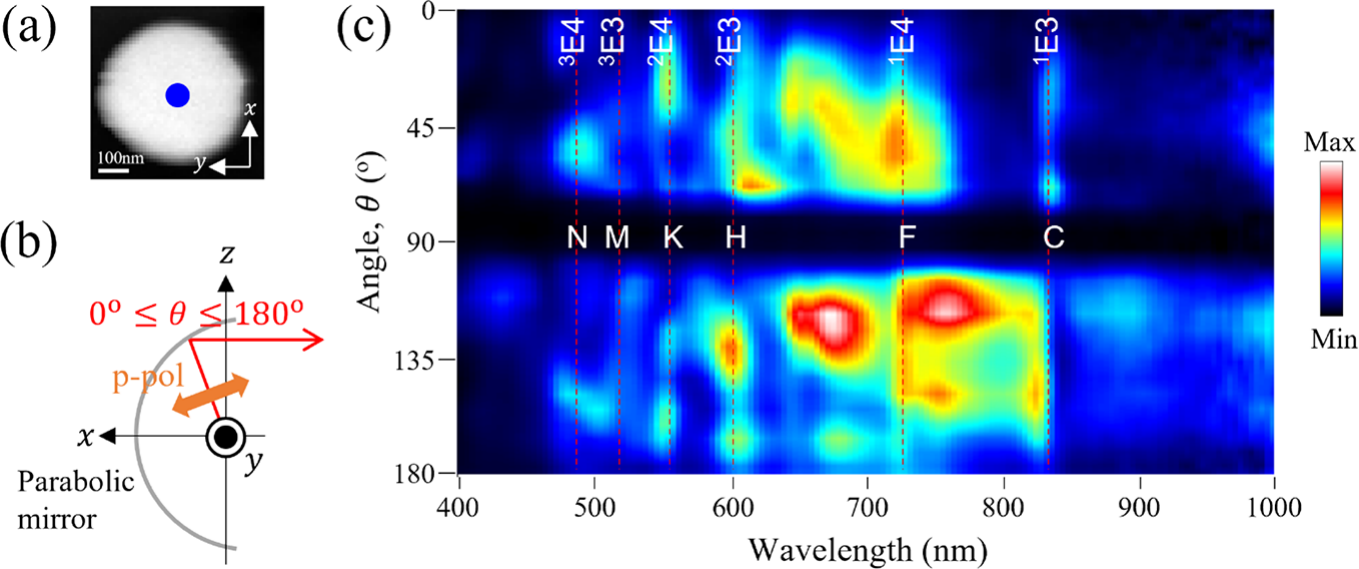
Angle-resolved spectrum (ARS) under electron excitation at the center of the particle. (a) STEM dark-field image of a Si sphere with a diameter of 490 nm. The blue dot indicates the position of the electron beam for central excitation. (b) Detection configuration of the measurement system for p-polarized light at emission angles 0^°^ ≤ θ ≤ 180^°^ with φ = 0^°^. (c) Experimentally obtained ARS pattern over the full range of detection angles θ under central electron-beam excitation.

To complement the analysis of the field distributions, studies on the angular emission of the mode interference at all detection angles θ are necessary, through angle-resolved spectrum (ARS) measurements that could reveal the directionality of the radiation from the excited modes. Figure 7 shows the ARS pattern with the excitation position placed at the center of the sphere, as indicated by a blue dot in Figure 7a, for p-polarized light collected at the detection angle θ that is scanned over the entire polar direction from 0^°^ to 180^°^, as shown in Figure 7b. The same 490 nm-diameter Si sphere as above is used in this measurement. The dark area around the horizontal angle θ = 90^°^ in the ARS pattern in Figure 7c is due to the shadowing effect of the sample support.

For central beam positioning, only the azimuthal *m* = 0 mode is allowed to be excited due to the axial alignment of the mode, in which rotational symmetry matches with the position of the electron beam.^26^ Further selecting p-polarized light emission, one restricts the detectable modes only to the electric ones. As a result, no significant radiation is detected from the magnetic ^1^M3 mode of peak #A at 973 nm and ^2^M1 mode of peak #B at 906 nm. Conversely, strong radiation is predicted to be emitted from the *m* = 0 electric modes. The broad spectral feature in the wavelength range of 650–900 nm is attributed to ^2^E2 and ^3^E1 modes (*m* = 0), as can be seen in the spectral mode contribution in Figure 3b and also in Figure S2b in the SI. In this broad emission band, rather sharp peak features are observed, such as the dominant electric ^1^E3 mode (*m* = 0) at peak #C. Moreover, the upward radiation (θ < 90^°^) of this #C resonance flips toward the downward direction (θ > 90^°^) on the shorter-λ side, an effect that can be attributed to interference with a broad feature of the lower-order electric mode with *m* = 0. The radiation flip is due to a phase flip of the sharp resonance. A similar radiation flip feature is also found around the peak #F, which can be attributed to the ^1^E3 mode. In addition, rather gradual radiation flips are also visible in the spectral range of 600–800 nm, corresponding to resonances produced by the ^2^E2 and ^3^E1 modes (see Figure 3b and Figure S2b in the SI).

Without the broad background feature, clear emission flips are not observed anymore. At resonance peak #H, radiation with a rather symmetric pattern on both the θ < 90^°^ and the θ > 90^°^ sides is attributed to the dominant electric ^2^E3 mode (*m* = 0). Similarly, a nearly even emission feature from the ^2^E4 mode (*m* = 0) is also observed at peak #K with stronger radiation coming mainly from the upper and lower lobes of the corresponding mode. Lastly, radiation distributions observed for peaks #M and #N over the angular θ range in the ARS pattern are attributed to *m* = 0 modes dominated by the electric ^3^E3 and ^3^E4 modes, respectively.

In summary, by fixing the electron beam at the center of the particle with a setup selecting p polarization, the azimuthal component *m* = 0 of the electric modes are selectively excited. Consequently, the distribution of features in the detected emission is observed to pile up around θ = 90^°^, as demonstrated in the ARS pattern. As for sphere-edge electron-beam excitation, we refer to the discussion of Figure S3 in the SI.

## Conclusions

We have presented experimental visualizations of the internal field distribution of whispering-gallery modes inside Si dielectric spheres (optical resonators) using a selective mode-extraction method of a 4D STEM-CL measurement system that allows for the simultaneous acquisition of angle- and wavelength-resolved four-dimensional mapping data. Our analysis reveals that the modes supported by Si spheres are strongly dependent on the size of the particles, for which higher-order modes emerge as the particle diameter increases, indicated by the presence of an increasing number of layered ring-shaped hotspots for emission at an angle θ = 180^°^. Motivated by this observation, one particle with a diameter of 490 nm was chosen for detailed analysis, in which we analytically found the presence of high-order modes at shorter wavelengths. Moreover, the fields from these excited modes for s-polarized light detected at an angle *θ* = 45^°^ are observed to approximately retain the main hotspot distribution of each respective dominant mode. In addition, p-polarized maps at a nearly horizontal angle θ = 80^°^ exhibit more complex features that are remarkably deviated from the base pattern of their most dominant modes due to strong interference with several other interfering modes. Simultaneous studies on angle-resolved maps demonstrate that the degenerate *m* modes can be resolved in the field map, which is dependent on the detection angle θ. Lastly, further analysis of the ARS pattern for central electron-beam excitation indicates that electric modes with *m* = 0 are selectively excited and that the radiation directionality flips due to the phase change around the resonances when two or more modes interfere. The distributions of such internal mode fields acquired through the field mapping technique introduced in our study can facilitate the design of applications relying on WGMs.

## Supporting Information Available

Identification of the WGM index in the WGM expansion, radiation distribution patterns, mode-resolved spectra, and ARS patterns under particle-edge electron-beam excitation (PDF)
