# Supplementary material for: Visualizing the Nanoscopic Field Distribution of Whispering-Gallery Modes in a Dielectric Sphere by Cathodoluminescence
Source: ACS Photonics. 2023 Mar 15;10(5):1434–45. doi: 10.1021/acsphotonics.3c00041 (PMC10197164; doi:10.1021/acsphotonics.3c00041)
Supplement: Supplementary file 1 — ph3c00041_si_001.pdf [file ph3c00041_si_001.pdf]

Supporting Information

Visualizing the Nanoscopic Field Distribution of Whispering-Gallery Modes in a Dielectric Sphere by Cathodoluminescence

Izzah Machfuudzoh[1], *Tatsuki Hinamoto*[2], *F. Javier García de Abajo*[3,4], *Hiroshi Sugimoto*[2], *Minoru Fujii*[2], *Takumi Sannomiya*[1,*]

[1] Department of Materials Science and Engineering, School of Materials and Chemical Technology, Tokyo Institute of Technology, 4259 Nagatsuta, Midori-ku, Yokohama, 226-8503 Japan

[2] Department of Electrical and Electronic Engineering, Graduate School of Engineering, Kobe University, Kobe 657-8501, Japan

[3] ICFO-Institut de Ciencies Fotoniques, The Barcelona Institute of Science and Technology, 08860 Castelldefels (Barcelona), Spain

[4] ICREA-Institució Catalana de Recerca i Estudis Avancats, Passeig Lluís Companys 23, 08010 Barcelona, Spain

Corresponding author

*Takumi Sannomiya (Email: sannomiya.t.aa@m.titech.ac.jp)

Identification of the WGM index

Direct identification of the whispering gallery mode (WGM) index is done through theoretical calculations that use appropriate approximations of the involved Bessel functions with an argument that considers a WGM near the sphere surface. Under this condition, the resonance positions for each field polarization P (i.e., either transverse electric (TE) or transverse magnetic (TM) modes) can be respectively obtained by solving the following equations[1]:

TE

$$Nx_{\ell,n}^{\mathrm{TE}} = \left(\ell + \frac{1}{2}\right) - \frac{1}{2^{1/3}}\left(\ell + \frac{1}{2}\right)^{\frac{1}{3}} \zeta_n - \frac{N}{\sqrt{N^2 - 1}} + \frac{1}{2^{2/3}}\frac{3}{10}\left(\ell + \frac{1}{2}\right)^{-\frac{1}{3}} {\zeta_n}^2 - \frac{1}{2^{1/3}}\left(\ell + \frac{1}{2}\right)^{-\frac{2}{3}} \frac{(-\frac{1}{3})N^3}{(N^2 - 1)^{3/2}} \zeta_n \tag{S1}$$

TM

$$Nx_{\ell,n}^{\mathrm{TM}} = \left(\ell + \frac{1}{2}\right) - \frac{1}{2^{1/3}}\left(\ell + \frac{1}{2}\right)^{\frac{1}{3}} \zeta_n - \frac{1/N}{\sqrt{N^2 - 1}} + \frac{1}{2^{2/3}}\frac{3}{10}\left(\ell + \frac{1}{2}\right)^{-\frac{1}{3}} {\zeta_n}^2 - \frac{1}{2^{1/3}}\left(\ell + \frac{1}{2}\right)^{-\frac{2}{3}} \frac{2 - 3N^4}{3N^3(N^2 - 1)^{3/2}} \zeta_n \tag{S2}$$

where N is the refractive index of the Si sphere[2], with the surrounding medium set as vacuum, and ζ_n denoting the nth pole of the Airy function[3] (i.e., $\mathrm{Ai}(\zeta) = 0$), which is associated with the radial mode order n. Regarding ℓ, it refers to the angular mode order, while x indicates the size parameter $x_{\ell,n}^{P} = 2\pi R/\lambda$ defined in terms of the sphere radius R and the resonance wavelength λ.

By applying the above Eqs. (S1) and (S2), the WGM index for each resonance peak of a Si sphere with a diameter of 490 nm is identified as indicated in the following table:

Table S1. WGM index identified through the calculated resonance wavelength positions.

Peaks	$\lambda_{\mathrm{experiment}}$ (nm)	λ_{WGM} (nm)	n	ℓ	P
**A**	973	973	1	3	TE
**B**	906	921	2	1	TE
**C**	831	819	1	3	TM
**D**	815	815	1	4	TE
**E**	755	756	2	2	TE
**F**	723	714	1	4	TM

**G1**	658	656	2	3	TE
**G2**	650				
**H**	606	588	2	3	TM
**I**	589	589	2	4	TE
**J**	569	570	3	2	TE
**K**	553	541	2	4	TM
**L**	528	524	3	3	TE
**M**	516	487	3	3	TM
**N**	479	463	3	4	TM

The results listed in Table S1 show that the measured experimental wavelengths ($\lambda_{\text{experiment}}$) for each peak are in good agreement with the calculated theoretical wavelengths for each WGM (λ_{WGM}), with an overall average percentage error of only around 1.5%. Additionally, the WGM index for each radial order n, angular momentum number ℓ, and field polarization P, which are identified directly in the WGM expansion (Table S1), are shown to match excellently with the same parameters n, ℓ, and P predicted in the multipole expansion, as indicated in the main text.

Radiation distribution pattern

The angular distribution of the radiation patterns can be calculated by using spherical harmonics[4] $\boldsymbol{Y}_{\ell,m}(\theta,\varphi)$, in term of which the electric field of the magnetic mode $\boldsymbol{H}_{\ell,m}(\theta,\varphi)$ and the electric mode $\boldsymbol{E}_{\ell,m}(\theta,\varphi)$ are written as follows[5]:

Magnetic mode
$$\boldsymbol{H}_{\ell,m}(\theta,\varphi) = \frac{1}{\sqrt{\ell(\ell+1)}}\vec{\boldsymbol{L}}\,\boldsymbol{Y}_{\ell,m}(\theta,\varphi) \quad \text{(S3)}$$

Electric mode
$$\boldsymbol{E}_{\ell,m}(\theta,\varphi) = \frac{1}{\sqrt{\ell(\ell+1)}}\vec{\boldsymbol{L}}\,\boldsymbol{Y}_{\ell,m}(\theta,\varphi) \times \hat{\boldsymbol{e}}_r \quad \text{(S4)}$$

where $\vec{\boldsymbol{L}}$ is the orbital angular momentum operator, while ℓ and m denote the total and azimuthal angular momentum numbers of the mode, respectively. The cross product with the radial unit vector $\hat{\boldsymbol{e}}_r$ in Eq. (S4) preserves the field orthogonality of magnetic and electric modes in a propagating electromagnetic wave. A detailed derivation of the field radiation can be found elsewhere[6].

Radiation distributions of multipolar modes with total angular momentum numbers extending from $\ell = 1$ to $\ell = 4$ and azimuthal numbers m in the range $|m| \leq \ell$ are plotted as follows:

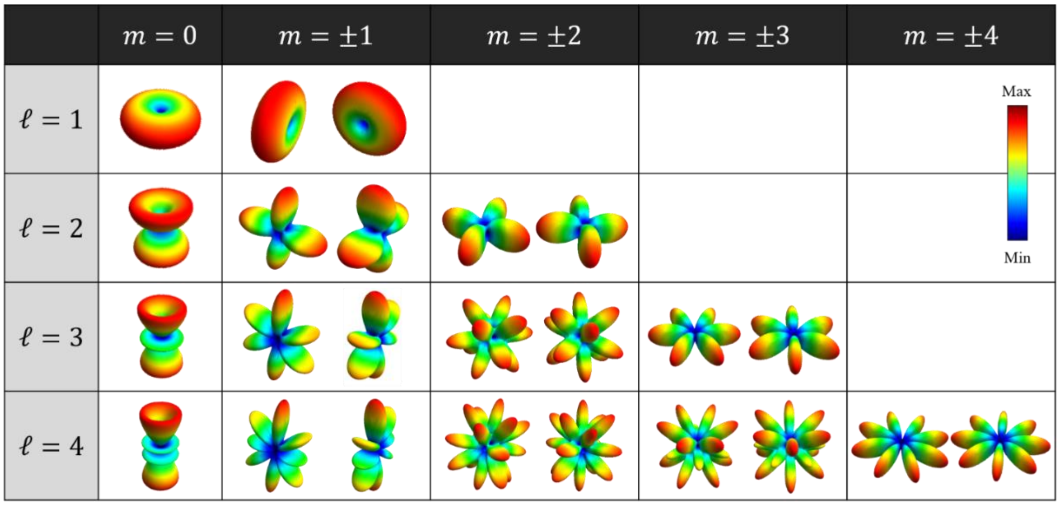

Figure S1. Angular plots of the radiation distribution for modes of low orders (ℓ, m).

Angle- and polarization-resolved spectra

Simulations of the angle- and polarization-resolved spectra for s-polarized light at an emission angle of $\theta = 45^{\mathrm{o}}$ and for p-polarized light at $\theta = 80^{\mathrm{o}}$, with a 490 nm-diameter Si sphere, are shown in Figs. S2a and S2b, respectively. Note that the most dominant modes in these detection conditions are the same as the ones in the integrated spectrum shown in Fig. 3b in the main text. The differences between these spectra are highlighted in the altered orders of their lower contributing dominant modes, indicating that some modes are indeed more pronounced in the contribution to the resulting mapping when the settings are narrowed down to a given angle and polarization. Moreover, we note that the experimental peaks #B at 906 nm and #J at 569 nm for s-polarized light at $\theta = 45^{\mathrm{o}}$ are not observed in the measured spectrum (Fig. 4c in the main text) when compared to the simulated one (Fig. S2a). This is probably due to the imperfect spherical shape of the Si particle, which compromises the detection of such fairly flat peaks in this configuration.

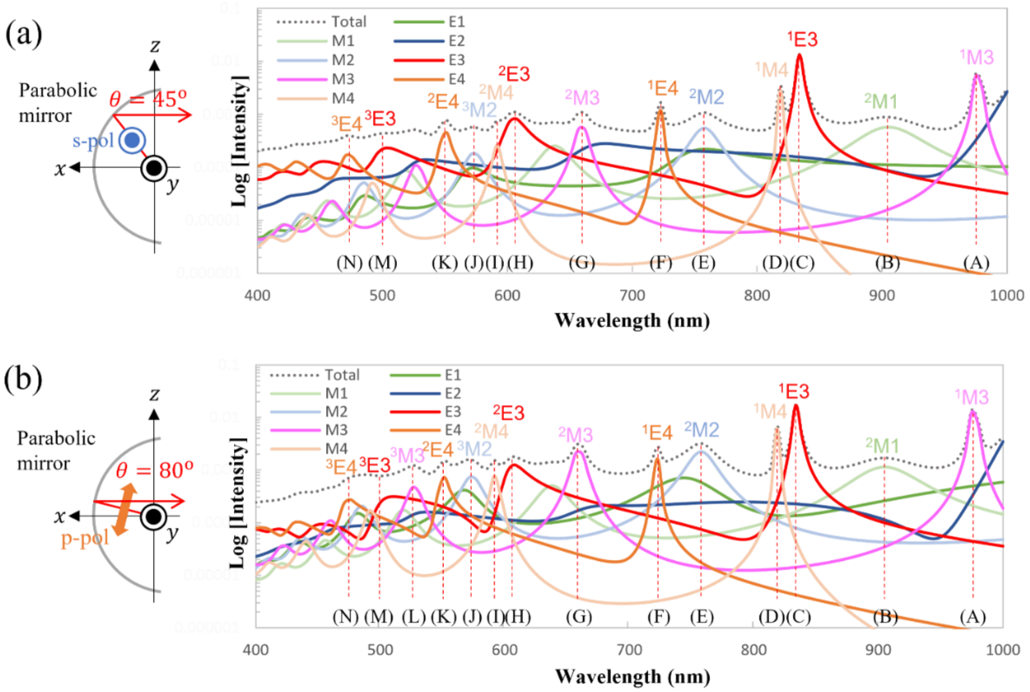

Figure S2. Detection configuration and simulated spectra of (a) s-polarized light collected at an angle $\theta = 45^{o}$, and (b) p-polarized light emitted at an angle $\theta = 80^{o}$. The dotted curve refers to the total spectrum, while the solid-colored curves correspond to the spectra of the extracted modes.

ARS pattern under sphere-edge excitation

The angle-resolved spectrum (ARS) pattern in the configuration of edge excitation with p-polarization is shown in Fig. S3. The electron beam is set at the bottom edge of the sphere, as shown by a blue dot in Fig. S3a. The emission peaks are indicated in the ARS pattern in Fig. S3c. The dark area that appears around $\theta = 90^{o}$ is produced by sample shadowing. The same Si sphere with a diameter of 490 nm as in the main text is used in this measurement.

Firstly, peak #A, which is dominated by a magnetic ^{1}M3 mode, is observed to have an emission mainly coming from the azimuthal orders $m = \pm 1$, as can be inferred by how the detected patterns in the ARS tend to spread out along the angle θ, indicating the radiation lobes of the mode with order $(\ell, m) = (3, \pm 1)$ (see Fig. S1). Peak #C also behaves similarly, with contributions from $m = \pm 1$ components of the dominant electric ^{1}E3 mode. As for peak #B, a faintly weak radiation

signal from the ^{2}M1 mode at only the downward side ($\theta > 90^{o}$) can be attributed to the interference with the quadrupole E2 mode ($m = \pm 2$) and the dipole E1 mode ($m = \pm 1$), analogously to the Kerker effect[7]. Moreover, regarding the weak emission associated with peaks #D and #E, the observed radiations are possibly contributed the most by $m = \pm 4$ components of the ^{1}M4 mode and by $m = \pm 1$ of ^{2}M2, respectively.

For peak #F, the edge-positioned electron beam dominantly excites $m = \pm 1$ components of the mode ^{1}E4, with some interference with other modes giving rise to strong downward emission. At the same time, peaks #G1 and #G2 in the ARS are observed to display more clearly separated spots that we mainly attribute to $m = \pm 1$ components of the dominant ^{2}M3 mode alone. A similar effect is also observed for the next peak #H, with a dominant electric ^{2}E3 mode ($m = \pm 1$) that overlaps with the radiation from the neighboring peak #I, which is in turn mostly contributed by the ^{2}M4 mode ($m = \pm 1$). The radiation distributions of the remaining peaks #J ($m = \pm 2$ of ^{3}M2), #K ($m = \pm 1$ of ^{2}E4), #L ($m = \pm 3$ of ^{3}M3), #M ($m = \pm 1$ of ^{3}E3), and #N ($m = \pm 2$ of ^{3}E4) can also be explained in a similar manner.

In brief, with the electron beam positioned at the particle edge, the excited modes are dominantly contributed by nonzero m-components of both electric and magnetic characters, in contrast to the results for central excitation, which only gives rise to $m = 0$ electric modes. This results in radiation distributed along all polar angles, including the $\theta = 0°$ and $180°$ directions.

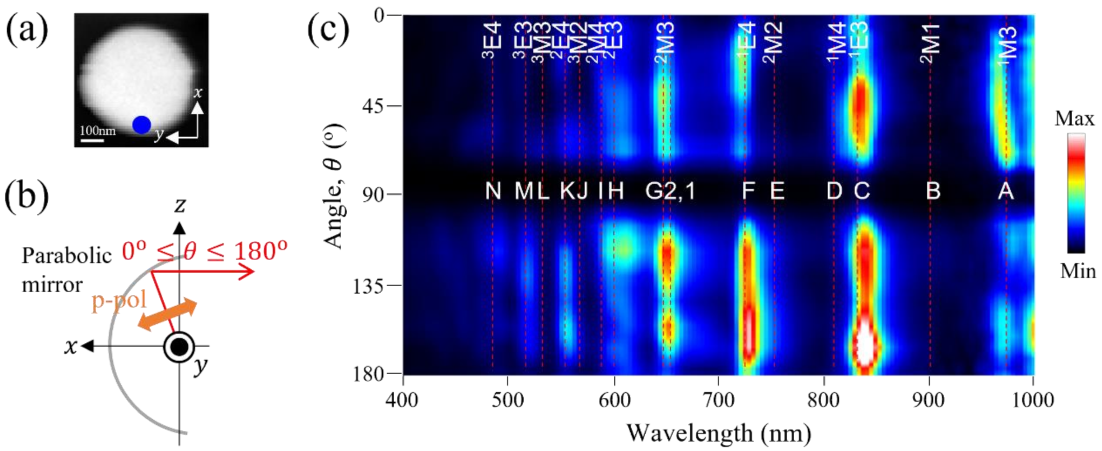

Figure S3. Angle-resolved spectrum (ARS) for electron excitation at the edge of the particle. (a) STEM image of a 490 nm-diameter Si sphere. The blue dot indicates the position of the electron beam for edge excitation. (b) Detection configuration of the measurement system for p-polarized light at an emission angle $(\theta, \varphi) = (\theta, 0^{o})$. (c) Measured ARS pattern over the full range of angles θ with bottom-edge excitation.

References

(1) Schiller, S.; Byer, R. L. High-resolution spectroscopy of whispering gallery modes in large dielectric spheres. *Optics Letters* **1991**, *16* (15), 1138-1140.

(2) Schinke, C.; Peest, P. C.; Schmidt, J.; Brendel, R.; Bothe, K.; Vogt, M. R.; Kröger, I.; Winter, S.; Schirmacher, A.; Lim, S.; et al. Uncertainty analysis for the coefficient of band-to-band absorption of crystalline silicon. *AIP Advances* **2015**, *5* (6), 067168.

(3) Abramowitz, M.; Stegun, I. A. *Handbook of Mathematical Functions: with Formulas, Graphs, and Mathematical Tables*; Dover Publications, 1972.

(4) Varshalovich, D. A.; Moskalev, A. N.; Khersonskiĭ, V. K. *Quantum theory of angular momentum irreducible tensors, spherical harmonics, vector coupling coefficients, 3nj symbols*; Singapore Hackensack, N.J World Scientific Pub. Co, 1988.

(5) Jackson, J. D. *Classical Electrodynamics*; John Wiley & Sons, 1998.

(6) Thollar, Z.; Wadell, C.; Matsukata, T.; Yamamoto, N.; Sannomiya, T. Three-Dimensional Multipole Rotation in Spherical Silver Nanoparticles Observed by Cathodoluminescence. *ACS Photonics* **2017**, *5* (7), 2555-2560.

(7) Kerker, M.; Wang, D.-S.; Giles, C. L. Electromagnetic scattering by magnetic spheres. *JOSA* **1983**, *73* (6), 765-767.
